# Pregnancy Zone Protein Is Associated with Airway Infection, Neutrophil Extracellular Trap Formation, and Disease Severity in Bronchiectasis

**DOI:** 10.1164/rccm.201812-2351OC

**Published:** 2019-10-15

**Authors:** Simon Finch, Amelia Shoemark, Alison J. Dicker, Holly R. Keir, Alexandria Smith, Samantha Ong, Brandon Tan, Jean-Yu Choi, Thomas C. Fardon, Diane Cassidy, Jeffrey T. J. Huang, James D. Chalmers

**Affiliations:** ^1^ Scottish Centre for Respiratory Research, Ninewells Hospital and Medical School, University of Dundee, Dundee, United Kingdom; and; ^2^ University of Cambridge, Cambridge, United Kingdom

**Keywords:** neutrophils, bronchiectasis, exacerbations, microbiome

## Abstract

**Rationale:** PZP (pregnancy zone protein) is a broad-spectrum immunosuppressive protein believed to suppress T-cell function during pregnancy to prevent fetal rejection. It has not previously been reported in the airway.

**Objectives:** To characterize PZP in the bronchiectasis airway, including its relationship with disease severity.

**Methods:** Label-free liquid chromatography/mass spectrometry was performed for sputum protein profiling of patients with bronchiectasis confirmed by high-resolution computed tomography. Results for patients with and without *Pseudomonas aeruginosa* infection were compared. Sputum and serum PZP was measured by validated ELISA. Airway infection status was established by culture and 16S ribosomal RNA sequencing. Immunofluorescence, ELISA, and electron microscopy were used to identify the cellular source of PZP in neutrophils treated with multiple stimuli.

**Measurements and Main Results:** Elevated PZP was identified by label-free liquid chromatography/mass spectrometry as being associated with *P. aeruginosa* infection. In a validation study of 124 patients, sputum but not serum concentrations of PZP were significantly associated with the Bronchiectasis Severity Index, the frequency of exacerbations, and symptoms. Airway infection with Proteobacteria such as *P. aeruginosa* was associated with higher concentrations of PZP. PZP in sputum was directly related to airway bacterial load. Neutrophils induced to form neutrophil extracellular traps (NETs) with phorbol myristate acetate released high concentrations of PZP *in vitro*, and fluorescence microscopy confirmed the presence of PZP in NETs, whereas fluorescence and electron microscopy localized PZP to the cytoplasm and nuclei of neutrophils. Effective antibiotic therapy reduced sputum PZP.

**Conclusions:** PZP is released into NETs. We report a novel link between airway infection, NET formation, and disease severity in bronchiectasis during chronic airway inflammation.

**At a Glance Commentary**

**Scientific Knowledge on the Subject**

Bronchiectasis is associated with chronic bacterial infection and neutrophilic inflammation. The underlying pathogenesis of the disease is poorly understood. Patients with chronic neutrophil inflammation have impaired innate and adaptive immunity, but the mechanisms by which neutrophilic inflammation links to impaired responses to infection are poorly characterized. In a proteomic study, we identified PZP (pregnancy zone protein), previously identified in the serum of pregnant women, in the airway of patients with severe bronchiectasis and *Pseudomonas aeruginosa* infection. PZP is a powerful T-cell immunosuppressant believed to prevent rejection of the fetal allograft during pregnancy. We hypothesized that PZP may be associated with airway infection susceptibility in bronchiectasis. In this study, we aimed to determine the source of PZP release in the airway and its association with chronic infection, airway inflammation, and disease severity.

**What This Study Adds to the Field**

We demonstrate, for the first time to our knowledge, that elevated airway concentrations of PZP are associated with disease severity, frequent exacerbations, and airway infection in patients with bronchiectasis. PZP was found in the cytoplasm of neutrophils and was released during acute and chronic pulmonary inflammation. PZP was found to be associated with neutrophil extracellular traps *in vitro* and correlated with neutrophil extracellular traps detected in bronchiectasis patient sputum *in vivo*. Our findings implicate NETosis in the pathophysiology of bronchiectasis, and given its known immunosuppressive effects, PZP may therefore provide a novel link between chronic neutrophilic inflammation and impaired host immunity to infection.

Bronchiectasis is a chronic respiratory disease characterized by lung inflammation, impaired mucociliary clearance, and recurrent airway infection leading to permanent tissue destruction and airway dilation. There is no licensed treatment for bronchiectasis, and therapeutic development has been severely limited by poor understanding of the pathogenesis of the disease (1). Bronchiectasis is a heterogeneous disease in terms of etiology, inflammatory profile, patient characteristics, comorbidities, and background treatments. Bronchiectasis is a global health problem, and further heterogeneity is added by differences in the aforementioned factors between different geographical regions (2–4). An apparent paradox in bronchiectasis is the persistence of pathogens in the airway despite the presence of a robust inflammatory response. During acute inflammation with gram-negative pathogens such as *Pseudomonas aeruginosa* or gram-positive pathogens such as *Staphylococcus aureus*, neutrophil recruitment is followed by phagocytosis of pathogens and clearance of both bacteria and neutrophils through apoptosis and efferocytosis by macrophages. During chronic inflammation, this process may be impaired with reduced phagocytosis, reduced neutrophil apoptosis, and a switch to tolerance and containment of infection through neutrophil extracellular trap (NET) formation. The mechanisms leading to this immunological tolerance remain largely unexplored in bronchiectasis (5).

PZP (pregnancy zone protein) is a high–molecular-weight glycoprotein that was originally described as being elevated in the serum of women during pregnancy (6). The synthesis of PZP is estrogen dependent, and it is detectable in serum a few weeks after conception and is reported to return to nearly undetectable concentrations immediately postpartum (7). PZP is a broad-spectrum immunosuppressant (8) with antiproteinase activity. Its role in pregnancy is believed to be suppression of cell-mediated immunoreactivity (9, 10) to prevent rejection of the fetus. PZP has been shown to depress T-lymphocyte immunoreactivity, T-cell recruitment, migration, proliferation, and IL-2 production (9). These immunosuppressive effects are profound, illustrated by a study showing that intravenous infusion of PZP was sufficient to prevent rejection of heart allografts in mice (11). Recent experiments using PZP knockout mice have shown that PZP also increases susceptibility to viral infection (12). PZP has never previously been found in the lung or studied in the context of chronic respiratory disease, however.

Chronic infection with *P. aeruginosa* is consistently associated with disease severity and poor outcomes in bronchiectasis. We therefore performed a proteomic study to profile sputum from patients with bronchiectasis and *P. aeruginosa* infection compared with those without chronic *P. aeruginosa* infection. Unexpectedly, PZP was identified as being elevated in patients with *P. aeruginosa* infection. We therefore hypothesized that, given the established immunosuppressive role of PZP, elevated sputum PZP would be associated with increased susceptibility to chronic airway infection and more severe disease.

Our results demonstrate, for the first time, to our knowledge, that PZP is released from neutrophils during degranulation and NET formation. PZP is associated with airway infection in patients with bronchiectasis and may be an unexpected mechanism through which NETs modulate T-cell function leading to increased susceptibility to respiratory infection.

## Methods

For detailed methods, please refer to the online supplement.

### Patients and Clinical Assessments

Patients were recruited at a specialist bronchiectasis clinic at Ninewells Hospital, Dundee, United Kingdom. Inclusion criteria were age 18 years or older, bronchiectasis confirmed by high-resolution computed tomographic scan, chronic expectoration with ability to provide a sputum sample at the study visit, and provision of written informed consent. Exclusion criteria were bronchiectasis due to cystic fibrosis, active allergic bronchopulmonary aspergillosis, active nontuberculous mycobacterial infection, chronic use of oral corticosteroids, a primary clinical diagnosis of another respiratory disease (chronic obstructive pulmonary disease [COPD] or asthma), and inability to provide informed consent. Ethical approval for the study was given by the East of Scotland Research Ethics Committee (approval number 12/ES/0059).

Severity of disease was evaluated using the Bronchiectasis Severity Index (BSI), as previously described (13). Exacerbations were defined as administration of antibiotics for increasing respiratory symptoms as defined by the British Thoracic Society (14). Sputum was obtained from all subjects during a period of clinical stability and split into whole (unprocessed) sputum for microbiology and sputum that was diluted 1:8 with phosphate-buffered saline and then centrifuged at 3,000 *g* for 15 minutes at 4°C. All sputum processing took place within 2 hours of expectoration, and freeze–thaw cycles were avoided.

### Sputum Protein Profiling

Sputum protein profiling was performed using nanoflow liquid chromatography/tandem mass spectrometry as previously described (15). Protein identification and label-free quantification were performed using MaxQuant software (https://www.maxquant.org/) (version 1.4.1.2) against the UniProt human database (version 2014-07-09; www.uniprot.org). The false discovery rate for protein identification was set to 1% at the protein level. Data visualization was performed using SIMCA-P software (version 13.0.3; Umetrics). For statistical analysis, the dataset was log_2_ transformed before being subjected to Student’s *t* test using Perseus software (https://maxquant.net/perseus/) (version 1.5.4.1). The Benjamini-Hochberg false discovery rate method was used, and corrected *P* values less than 0.05 were considered significant.

### PZP ELISA

PZP was measured using a commercial ELISA kit for human and mouse (Cloud-Clone Corp.). Validation was performed according to published recommendations (16).

### Quantification of Sputum NETs

Measurement of histone–elastase and DNA–elastase complexes provides a semiquantitative assessment of NETs in sputum, and assays were performed as previously described (17).

### Leukocyte Studies in Healthy Volunteers

Neutrophils and peripheral blood monocytes were isolated from healthy volunteers using Percoll gradient density centrifugation as previously described (18). Immunofluorescence was used to confirm and localize PZP within neutrophils and NETs and to identify colocalization with other neutrophil proteins. NET formation was induced by treatment for 4 hours with 600 nM phorbol myristate acetate (PMA).

Colocalization of PZP with neutrophil granule proteins was quantified using the Manders overlap coefficient, which calculates the proportion of overlap of each channel with the other, with a value of 1 indicating perfect colocalization and 0 indicating no colocalization.

Electron microscopy was used to identify the cellular location of PZP after staining with anti-PZP antibody and Nanogold secondary antibody (Nanoprobes Inc.). Appropriate negative controls were included.

### Murine Model of Acute Inflammation

Female 10–12-week-old C57BL/6 mice were infected with *S. aureus* strain RN6390 at an infecting dose of 3 × 10^8^ cfu. At 24 hours after infection, the trachea was carefully dissected and intubated, and BAL was performed with three instillations of 0.4 ml of phosphate-buffered saline. BAL supernatant was used for PZP quantification and cells for cytospins to quantify neutrophils.

### Sputum Bacteriology

Quantitative bacterial culture was performed as described in the online supplement.

### Microbiota Sequencing

PCR and sequencing of the 16S ribosomal (r)RNA gene were performed on an Illumina MiSeq system as previously described (17). Analysis was performed in Quantitative Insights Into Microbial Ecology (detailed methods in online supplement). α Diversity was evaluated using the Shannon-Wiener diversity index and the Berger-Parker index. To compare groups, patients were split into those with predominant (>50% operational taxonomic units [OTUs]) Proteobacteria and those with predominant Firmicutes at the phylum level, as previously described.

### Antibiotic Response Study

Patients were asked to attend the research center if they developed symptoms of an exacerbation. Patients were reviewed by a physician, and those who were prescribed antibiotics for a protocol-defined exacerbation were included in the study. Patients received treatment for 14 days on the basis of their previous sputum microbiology. Spontaneous sputum samples obtained as baseline and after 14 days were used for PZP measurement.

### COPD Cohort Study

To compare sputum PZP concentrations in bronchiectasis with those from patients with COPD, 40 patients with COPD without underlying bronchiectasis were enrolled while clinically stable (4 wk free from antibiotic or corticosteroid therapy). Spontaneous sputum samples were obtained and processed in the same way as the bronchiectasis samples with sputum PZP and sputum NETs measured by ELISA.

### Statistical Analysis

Statistical analysis was performed using Prism version 8 software (GraphPad Software). Categorical variables are presented as frequencies and percentages, and statistical differences were analyzed using the χ^2^ test or Fisher’s exact test when required. Continuous variables are presented as mean and SD or median and interquartile range (IQR) when data are not normally distributed. Sputum PZP was not normally distributed, so data were logarithmically transformed and then analyzed using Student’s *t* test for comparisons of two groups and one-way ANOVA for more than two groups. A paired Student’s *t* test was used to compare changes in sputum PZP with antibiotic therapy. Linear variables were correlated by Spearman correlation. Principal component analysis (with the dataset logarithmically transformed, mean centered, and unit variance scaled) was performed using SIMCA-P version 13.0.3 software. We defined statistical significance as a two-tailed *P* < 0.05 for all analyses.

## Results

**Figure 1.**
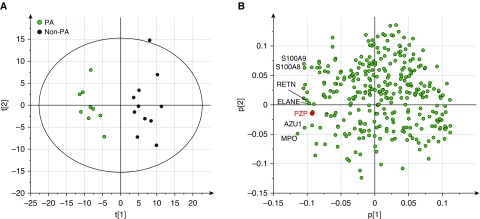
Principal component analysis of sputum protein profiles in bronchiectasis. Twenty patients with bronchiectasis, including nine with *Pseudomonas aeruginosa* (PA) infection (labeled in green) and eleven without (labeled in black), were included. (*A* and *B*) The scores plot based on the first two components is shown in *A*, and the loadings plot is shown in *B*. The cumulative R2X = 0.31 and Q2 = 0.21. R2X represents the fraction of the variation of the X variables explained by the first two components, and Q2 indicates the fraction of the variation of the X variables predicted by the model. Sputum PZP (pregnancy zone protein) concentrations are associated with samples with *P. aeruginosa* infection.

Sputum protein profiling was performed in 20 patients with bronchiectasis (9 with *P. aeruginosa* infection and 11 without) to explore potential biomarkers relevant to disease severity in bronchiectasis. Characteristics of the patients included are shown in Table E1 in the online supplement. Principal component analysis of sputum protein profiles revealed two distinct clusters in which sputum profiles of patients with *P. aeruginosa* are well separated from those without (Figure 1A). A total of 80 proteins were found to be significantly associated with *P. aeruginosa* in sputum. Among differentially expressed proteins, many were previously identified biomarkers of bronchiectasis lung disease, including neutrophil elastase, myeloperoxidase, S100-A8, and S100-A9, as shown in the loadings plot in Figure 1B. We identified PZP, not previously described as present in sputum or as a neutrophil-associated protein, to be differentially expressed between samples with *P. aeruginosa* infection and those without. Direct comparison of PZP in the *P. aeruginosa* infected and uninfected groups is shown in Figure E1. The raw proteomics dataset has been uploaded as Table E4. We therefore explored whether PZP was associated with neutrophilic inflammation and bronchiectasis disease severity.

### Patient Cohort

One hundred twenty-four patients were included in the study. There was a slight female predominance and a mean age of 67 years, typical of European bronchiectasis cohorts (19–21). The clinical characteristics of our cohort are shown in Table 1.

**Table 1.** Patient Characteristics

Characteristics	Data
*N*	124
Age, yr, mean (SD)	69.1 (10.7)
Sex, F, %	53.2
BSI	
Mean (SD)	7.8 (4.2)
Mild, *n* (%)	30 (24.2)
Moderate, *n* (%)	56 (45.2)
Severe, *n* (%)	38 (30.6)
Exacerbation, frequency/yr, mean (SD)	2.6 (2.1)
FEV_1_% predicted, mean (SD)	78.1 (25.5)
Etiology, *n* (%)	
Idiopathic	82 (66.1)
COPD	16 (12.9)
Postinfective	8 (6.5)
Inflammatory bowel disease	4 (3.2)
Immunodeficiency	3 (2.4)
ABPA*	2 (1.6)
Rheumatoid arthritis	2 (1.6)
PCD	1 (0.8)
Aspiration	1 (0.8)
Young syndrome	1 (0.8)
Asthma	1 (0.8)
GERD	1 (0.8)
Hematological malignancy	1 (0.8)
Other	1 (0.8)
Smoking, *n* (%)	
Current smoker	12 (9.7)
Ex-smoker	52 (41.9)
Never smoker	60 (48.4)
Antibiotics, *n* (%)	
Long-term macrolides	27 (21.8)
Inhaled	5 (4)

*Definition of abbreviations*: ABPA = allergic bronchopulmonary aspergillosis; BSI = Bronchiectasis Severity Index; COPD = chronic obstructive pulmonary disease; GERD = gastroesophageal reflux disease; PCD = primary ciliary dyskinesia.

^*^ These patients had previously treated ABPA rather than active disease.

### Association of Sputum PZP with Disease Severity in Bronchiectasis

The median serum concentration of PZP was 4.1 μg/ml (IQR, 2.2–9.9), consistent with published data describing concentrations expected in healthy men and women (<10 μg/ml in men and postmenopausal women, 10–30 μg/ml in premenopausal women) (22, 23). The median sputum concentration was 65.9 μg/ml (IQR, 36.9–205.8). There was no significant difference in sputum PZP between male and female patients (median, 65 μg/ml [IQR, 39.1–215] vs. 66.8 [IQR, 34.7–198.5] μg/ml; *P* = 0.8). There was no significant correlation between serum and sputum PZP concentrations (*see* Figure E2).

**Figure 2.**
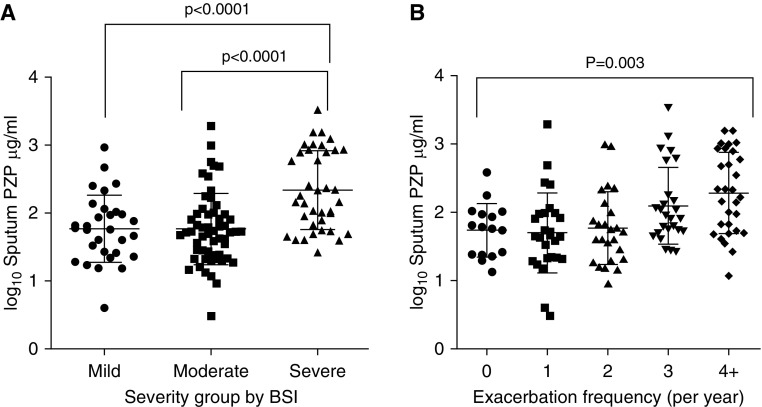
Association between sputum PZP (pregnancy zone protein) and clinical outcomes. (*A*) Severity of disease as stratified by Bronchiectasis Severity Index (BSI). (*B*) Exacerbation frequency in previous year.

Using the validated BSI, we observed a clear relationship between sputum PZP and bronchiectasis severity. There was a significant elevated median sputum PZP in patients with severe disease of 163 μg/ml (IQR, 64.6–854.1) compared with patients with mild disease (58.6 μg/ml; IQR, 25.3–163.8) or those with moderate disease (52.6 μg/ml; IQR, 24.1–97.3) (*P* < 0.001) (Figure 2A). It was observed that sputum PZP concentrations were not normally distributed, so data are displayed as log_10_ values.

Sputum PZP was also higher in patients with frequent exacerbations (≥3/yr) than in patients with less frequent exacerbations, as shown in Figure 2B. Relationships were also observed with quality of life using the Quality of Life Questionnaire–Bronchiectasis respiratory symptom score (*P* < 0.0001), and a weaker association was observed with FEV_1_ percent predicted (*r* = −0.21; *P* = 0.02). Patients with higher PZP sputum concentrations also had a higher daily sputum volume (*r* = 0.2; *P* = 0.02), whereas hospitalization for a severe exacerbation was also predicted by higher sputum PZP (*P* < 0.0001). No significant associations were observed between serum PZP and severity of disease (Figure E3).

### PZP Is a Marker of Airway Chronic Infection

**Figure 3.**
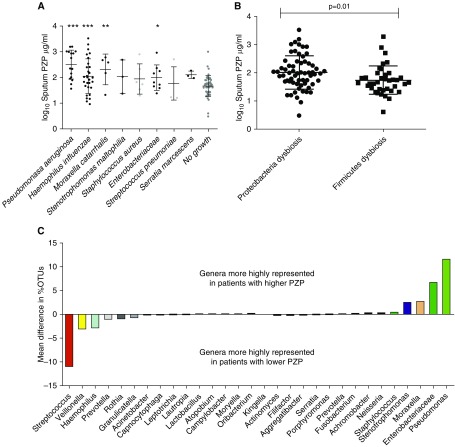
Association between sputum PZP (pregnancy zone protein) and microbiological outcomes. (*A*) Sputum PZP by microorganism growth on standard culture. (*B*) Sputum PZP by dysbiosis (defined as >50% reads of single phylum on 16S sequencing). (*C*) Patients were divided into those with PZP above and below the median value for the population, and the percentage operational taxonomic units (OTUs) were compared. The 15 genera most strongly associated with lower PZP concentrations and the 15 genera most strongly associated with higher PZP concentrations are shown. *P < 0.05, **P < 0.001, and ***P < 0.0001.

Airway infection is linked to disease severity, and we next validated the previous observation that PZP concentrations are higher in patients with chronic airway infection. Using standard microbial culture, we found that the most frequently isolated pathogens were *Haemophilus influenzae* (*n* = 29) and *P. aeruginosa* (*n* = 16). Overall, 75 patients had chronic infection with pathogens, and 49 did not. As shown in Figure 3A, patients with *P. aeruginosa* infection had significantly higher concentrations of sputum PZP than patients with no growth of pathogens. *H. influenzae*, *Moraxella catarrhalis*, and *Enterobacteriaceae* were also associated with higher concentrations of sputum PZP.

When we assessed bacterial diversity by 16S rRNA sequencing, we observed no relationship between sputum PZP and α diversity using either the Shannon-Wiener diversity index or the Berger-Parker index. PZP concentrations were significantly different in patients with different microbiota profiles at the phylum level, however, with those with Proteobacteria dysbiosis (defined as >50% reads) having significantly higher sputum PZP (*P* = 0.01) (Figure 3B). Patients with PZP concentrations above the median of the population had a higher average percentage of OTUs classified as *Pseudomonas* and a lower percentage of OTUs classified as *Streptococcus* and *Veillonella* (Figure 3C).

### Sputum PZP Is Related to Airway Bacterial Load and Is Reduced by Antibiotic Therapy

**Figure 4.**
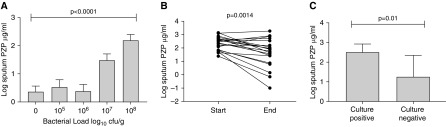
Association between bacterial load in sputum and PZP (pregnancy zone protein). (*A*) Sputum bacterial load is associated with sputum PZP (*P* value refers to comparison by ANOVA). (*B*) Changes in PZP at the start (Day 0) and end (Day 14) of antibiotic therapy for an acute exacerbation of bronchiectasis. The *P* value refers to comparison by paired Student’s *t* test. (*C*) Comparison of patients in *B* with positive or negative cultures at the end of antibiotic treatment for an exacerbation. *P* value refers to unpaired Student’s *t* test.

Bacterial load was quantified in 60 patients with bronchiectasis. The characteristics of this subgroup are shown in the online supplement (Table E2). PZP concentrations were significantly associated with increased bacterial load (*P* < 0.0001 by ANOVA (Figure 4A). Significant differences were observed between those with bacterial loads above 10 (7) and all other subgroups (*P* < 0.05 for all pairwise comparisons). Excluding patients infected with *P. aeruginosa* produced similar results (Figure E4).

Reduction in bacterial load with antibiotic treatment of exacerbations was associated with reduced sputum PZP concentrations. Twenty patients were treated, 18 of whom had detectable bacterial loads at baseline. Figure 4B shows the changes in sputum PZP from baseline to the end of treatment after 14 days. PZP was significantly reduced (*P* = 0.0014 by paired *t* test). Six patients, five of whom had *P. aeruginosa* infection, had detectable bacterial loads despite 14 days of antibiotics. Those patients who remained culture positive after 14 days had higher PZP at the end of treatment than those who achieved bacterial clearance (*P* = 0.01) (Figure 4C). Taken together, these data show that PZP increases with increasing bacterial load, regardless of the infecting pathogen, and that reducing bacterial load with antibiotic therapy reduces sputum PZP.

### PZP Is Released from Neutrophils during Activation and Acute Infection

**Figure 5.**
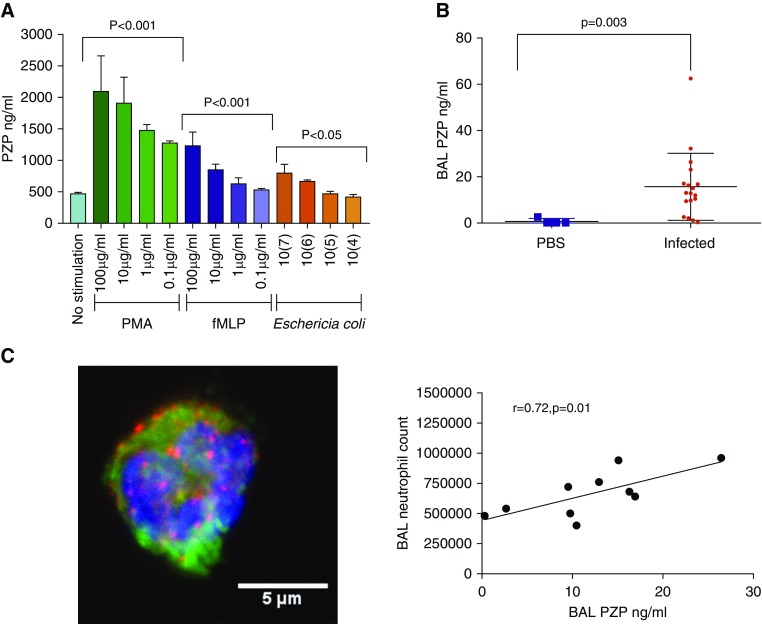
PZP (pregnancy zone protein) is released from neutrophils in response to formylmethionylleucylphenylalanine (fMLP), phorbol myristol acetate (PMA), and bacterial infection in mice and humans. (*A*) Neutrophils (*n* = 10^7^) were treated with the indicated stimuli for 30 minutes, and PZP was measured in cell-free supernatants. The no-stimulation control was phosphate-buffered saline (PBS) only. Statistically significant differences were obtained using Student’s *t* test (*n* = 4 biological replicates per condition). (*B*) C57BL/6 mice aged 10–12 weeks were infected with *Staphylococcus aureus* strain RN6390 (*n* = 18) or PBS control (*n* = 4). BAL PZP was measured by ELISA. Significant differences were determined using Student’s *t* test. An association was found between BAL neutrophils and BAL PZP (*n* = 10 mice undergoing BAL, all infected with *S. aureus*). (*C*) Microscopic image of neutrophils showing DNA. Blue = DAPI; green = neutrophil elastase; red = PZP.

We next examined the potential source of PZP detected in the bronchiectasis airway. There was no detectable PZP in the supernatants or cell lysates of primary human bronchial epithelial cells. Mononuclear cells released small amounts of PZP into the supernatant (mean, 46.7 ng/ml; SD, 18.2; *n* = 4), with higher concentrations seen upon stimulation with 2.5 ng/ml PMA (mean, 177.8 ng/ml; SD, 6.4; *n* = 4). Neutrophils secreted large amounts of PZP when stimulated with formylmethionylleucylphenylalanine and PMA (Figure 5A). A dose-dependent relationship was noted with all stimulants. PZP was also released after incubation with bacteria (*Escherichia coli* and *P. aeruginosa*) in a dose- and time-dependent manner (Figures 5A and E5). Immunofluorescence by confocal microscopy confirmed the presence of PZP in neutrophils (Figures 5B, E6, and E7 and Videos E1 and E2). Staining was seen in a diffuse, punctate pattern throughout the cytoplasm, possibly suggestive of granules; however, significant colocalization with other granule proteins (neutrophil elastase, lactoferrin, myeloperoxidase, and matrix metalloproteinase 9) was not observed (Manders overlap coefficient, <0.3).

We studied whether acute pulmonary infection, which provokes a neutrophil-mediated inflammatory response, would result in an increase in PZP in BAL. *S. aureus* was used as a common bronchiectasis pathogen that provokes a robust neutrophil response, including the formation of NETs. At 24 hours after infection, *S. aureus* was detected in lung homogenates of infected mice, and this was associated with BAL neutrophilia. As shown in Figure 5C, infection resulted in an increase in PZP in BAL that was directly correlated to BAL neutrophil count.

### PZP Is Found in the Nucleus and Cytoplasm of Neutrophils and Released into NETs

**Figure 6.**
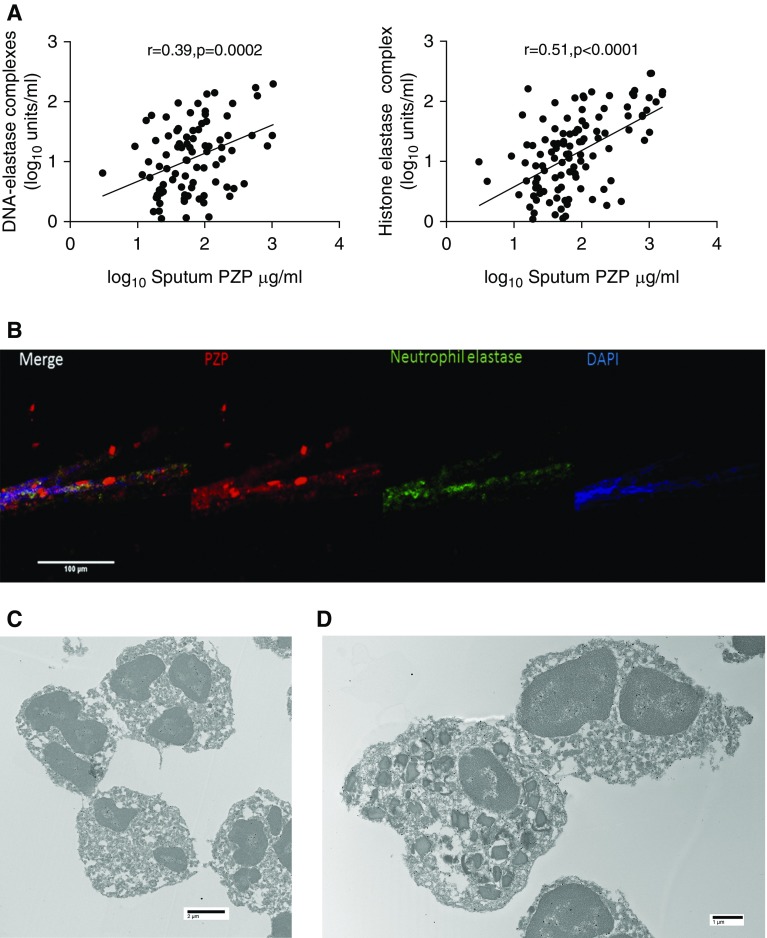
PZP (pregnancy zone protein) is a cytoplasmic and nuclear protein that is released in neutrophil extracellular traps (NETs). (*A*) In patients with bronchiectasis, extracellular PZP correlates with markers of NET formation. (*B*) Peripheral blood neutrophils were induced to undergo NET formation with 600 nM phorbol myristol acetate for 4 hours. The NETs contain PZP. (*C* and *D*) Electron microscopy of neutrophils (*C*) and neutrophils plus eosinophils (*D*) showing diffuse cytoplasmic and nuclear staining for PZP (black dots).

On the basis of the observation that PMA, an inducer of NET formation, was a strong stimulus for PZP release from neutrophils, we hypothesized that PZP may be a marker of NETosis. In bronchiectasis sputum *in vivo*, we identified a strong correlation between PZP in sputum and NETs measured by histone–elastase or DNA–elastase complexes (Figure 6A). Experimentally induced NETs from healthy control neutrophils treated with PMA showed staining for PZP in association with DNA and neutrophil elastase in a “studded” pattern typical of NETs (Figure 6B). To further investigate the localization of PZP in neutrophils, we used immune electron microscopy. This revealed staining within the euchromatin of the nuclei and a diffuse cytoplasmic pattern of PZP within the neutrophil. Our granulocyte extraction method produces neutrophil preparations that are >95% pure but may contain small numbers of eosinophils. We unexpectedly observed positive staining of eosinophils in a similar pattern to neutrophils with cytoplasmic and nuclear staining.

**Figure 7.**
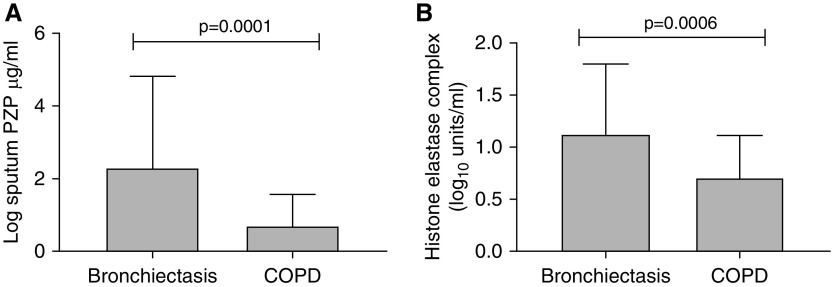
(*A*) PZP (pregnancy zone protein) concentrations in sputum in patients with bronchiectasis (*n* = 124) or chronic obstructive pulmonary disease (COPD) (*n* = 40). (*B*) Neutrophil extracellular traps measured using the histone–elastase complex assay in bronchiectasis and COPD. For both assays, comparisons are by Student’s *t* test.

Bronchiectasis is not alone in causing chronic neutrophilic inflammation of the airway. COPD is characterized by variable degrees of both neutrophilic and eosinophilic inflammation, and NETs have been reported in the COPD airway. We therefore measured PZP and NETs in sputum from patients with COPD during a period of stability and compared these with samples obtained from our bronchiectasis cohort. Characteristics of the subjects with COPD are shown in Table E3. Both PZP and NETs were significantly lower in subjects with COPD than in those with bronchiectasis (Figure 7).

## Discussion

This study has identified PZP, which was previously described as a serum protein elevated in the blood of pregnant women, as an unexpected component of NETS. It was found to be released into the bronchiectasis airway during chronic infection, and it is elevated in patients with the most severe disease and frequent exacerbations.

We found concentrations of PZP in sputum at least 10 times higher than concentrations in serum, suggestive of local production by inflammatory cells in the airway. It is known that patients with more severe bronchiectasis have higher degrees of airway neutrophilic inflammation (24), including markers such as neutrophil elastase (25), matrix metalloproteinases (26, 27), and cathelicidin (28). PZP followed a similar pattern with clear associations with the multidimensional BSI, a higher concentration in the “frequent exacerbator phenotype,” and an association with respiratory symptoms.

The major driver of airway neutrophilic inflammation in bronchiectasis is bacterial infection according to the vicious vortex concept of bronchiectasis pathophysiology. We demonstrate that PZP is increased in patients with chronic infection, particularly with *P. aeruginosa* and *H. influenzae*, both frequent pathogens in this disease. Molecular “microbiome”–based analysis of sputum confirmed elevated PZP in patients with Proteobacteria dysbiosis and airway dominance of *Pseudomonas*. This correlates with the clinically observed phenotypes in which patients who regularly culture Proteobacteria (predominantly *Pseudomonas* and *Haemophilus*) display clinically more severe phenotypes (29).

We demonstrate that neutrophils are the likely source of PZP, although we found that, in common with many immune proteins, PZP was present in a number of cell types, including monocytes and eosinophils. PZP appears to be present throughout the cytoplasm of neutrophils and is not concentrated within primary or secondary granules; it is also consistently visible within the nuclei of both neutrophils and eosinophils. We demonstrate in a mouse model of *S. aureus* pneumonia that PZP is released into BAL after infection in parallel with neutrophil influx.

Neutrophils eliminate pathogens through phagocytosis, a relatively noninflammatory intracellular process, and extracellularly through degranulation. NET formation is a distinct antimicrobial pathway in which neutrophils can extrude extracellular DNA, histones, and bactericidal proteins intended to trap and neutralize pathogens. Components released in NETs include antimicrobial peptides (lactoferrin, defensins, LL-37, and bacterial permeability–increasing protein), proteases (neutrophil elastase, proteinase 3, and gelatinase), and enzymes responsible for reactive oxygen species generation (myeloperoxidase). NETs have been described in many chronic diseases, including chronic respiratory diseases such as COPD and cystic fibrosis. It is believed that they serve a beneficial role in preventing spread of bacterial infection but also contribute to tissue damage.

The identification of PZP in NETs in bronchiectasis is intriguing because it has no known antimicrobial effects. The known effects of PZP are as an antiproteinase and as a powerful T-cell immunosuppressant. We speculate PZP may be involved in immunological tolerance in patients with bronchiectasis by interacting with NETs to modulate T-cell functions. The persistence of bacterial infection despite an apparently intact cell-mediated immune system is a feature of bronchiectasis that remains unexplained. It is well established that both gram-positive and gram-negative organisms can induce NET formation, and it has been shown that neutrophils undergo NET formation in circumstances in which phagocytosis is prevented, such as with physical barriers preventing phagocytosis, as may occur with biofilms or with high bacterial loads. We demonstrated a strong association between PZP and elevated airway bacterial load above 10^7^ cfu/g, suggesting that NET formation may be the dominant neutrophil phenotype in patients with high bacterial loads. Antibiotic therapy was able to reduce PZP concentrations consistent with a cause-and-effect relationship between bacterial infection and elevated PZP.

The positive staining of eosinophils in a similar pattern to neutrophils is of interest. The inflammation in bronchiectasis is a predominantly neutrophilic process, but eosinophilic phenotypes do exist.

Multiple previous reports indicate that PZP is regulated by estrogens and other female reproductive hormones, but we found no evidence of a sex-based difference in serum or sputum PZP concentrations. Bronchiectasis has a female predominance, but our evidence suggests that PZP is unlikely to play any role in this sex disparity. Both the male and female patient groups in our cohort are elderly. It has been shown that the physiological fluctuations in estrogen concentration affect exacerbations and *P. aeruginosa* mucoid transformation in cystic fibrosis (30), but such fluctuations will not occur in a predominantly postmenopausal cohort. Testosterone has also been shown to affect the antimicrobial susceptibility of *P. aeruginosa* and has established roles in host defense (31). Future studies are required to define how sex hormones may be implicated in the pathogenesis of bronchiectasis, but we found no evidence in established disease that PZP was associated with sex differences.

In summary, this study has identified a novel neutrophil protein that is released predominantly from neutrophil cytoplasm into NETs during chronic inflammation. We demonstrate high concentrations of PZP in the airway in severe disease and in patients with high bacterial loads. These data suggest, for the first time, to our knowledge, that NETs are present in the bronchiectasis airway, are associated with disease severity, and contain proteins with the potential to have profound effects on the innate and adaptive immune system. The validation of this observation in multiple datasets using proteomics and ELISA suggests that this finding is robust. Bronchiectasis is a heterogeneous disease, and there is increasing interest in identifying phenotypes and endotypes with distinct clinical outcomes and treatment responses. The finding that PZP is a marker of neutrophil-mediated inflammation may be important for other diseases in which neutrophils play a crucial role. Further mechanistic work is required to determine whether PZP is simply a marker of chronic neutrophilic inflammation or if it has a direct role in the pathogenesis of chronic infection in bronchiectasis.

The strength of this study is the use of multiple methods of clinical assessment and two different cohorts to validate our findings, as well as the confirmation of the finding of PZP in neutrophils using multiple methods, including ELISA, immunofluorescence, and electron microscopy. Nevertheless, the study has limitations. There are no animal models of bronchiectasis, so there is no direct method of testing whether PZP is directly involved in the pathogenesis of bronchiectasis. We used an acute model of pulmonary inflammation with *S. aureus* because this organism promotes a robust neutrophil-mediated response, but we acknowledge that alternative models, such as models of chronic *P. aeruginosa*, may provide complementary information.

In conclusion, this study has identified a novel protein in the bronchiectasis airway associated with NETs, chronic infection, and disease severity.

## Supplementary Material

Supplements

Author disclosures
