# Supplementary material for: Pregnancy Zone Protein Is Associated with Airway Infection, Neutrophil Extracellular Trap Formation, and Disease Severity in Bronchiectasis
Source: Am J Respir Crit Care Med. 2019 Oct 15;200(8):992–1001. doi: 10.1164/rccm.201812-2351OC (PMC6794104; doi:10.1164/rccm.201812-2351OC)
Supplement: Supplements [file rccm.201812-2351OC.html]

Pregnancy Zone Protein Is Associated with Airway Infection, Neutrophil Extracellular Trap Formation, and Disease Severity in Bronchiectasis | American Journal of Respiratory and Critical Care Medicine

- disclosures.pdf (4 MB)
- finch\_data\_supplement.pdf (1 MB)
- finch\_supplemental\_table e4.xlsx (140 KB)
- finch\_supplemental\_video\_e1.avi (294 KB)
- finch\_supplemental\_video\_e2.avi (145 KB)
