# Supplementary material for: Pregnancy Zone Protein Is Associated with Airway Infection, Neutrophil Extracellular Trap Formation, and Disease Severity in Bronchiectasis
Source: Am J Respir Crit Care Med. 2019 Oct 15;200(8):992–1001. doi: 10.1164/rccm.201812-2351OC (PMC6794104; doi:10.1164/rccm.201812-2351OC)
Supplement: Supplements [file rccm.201812-2351OC_finch_data_supplement.pdf]

ONLINE SUPPLEMENT FOR:

Pregnancy Zone Protein is Associated with Airway Infection, Neutrophil Extracellular Trap Formation and Disease Severity in Bronchiectasis

Simon Finch, Amelia Shoemark, Alison J Dicker, Holly R Keir, Alexandria Smith, Samantha Ong, Brandon Tan, Jean-Yu Choi, Thomas C Fardon, Diane Cassidy, Jeffrey TJ Huang, James D Chalmers

Corresponding author: Prof James D Chalmers, Scottish Centre for Respiratory Research, University of Dundee, Ninewells Hospital and Medical School, Dundee, DD1 9SY, UK. E-mail: jchalmers@dundee.ac.uk

Subject category: 10.16: Non-cystic fibrosis bronchiectasis

Key-words: Neutrophils; bronchiectasis; exacerbations; microbiome, biomarker.

Supported by: Funded by the Scottish Government Chief Scientist Office (Clinical Academic Fellowship to SF and Senior Clinical Fellowship to JDC) and by the British Lung Foundation through the GSK/British Lung Foundation Chair of Respiratory Research.

Supplementary results

	Pseudomonas, n=9	Non-Pseudomonas, n=11
Age (mean)	63	66.91
Sex	66.6% female (n=6)	54.5% female (n=6)
SGRQ score	57.8	45.25
Previous hospitalisations (mean)	0.89	0.27
Exacerbations since last visit (mean)	4.78	1.91
MRC dyspnoea score	3.11	1.91

Table E1. Characteristics of the patients in original proteomics screening study.

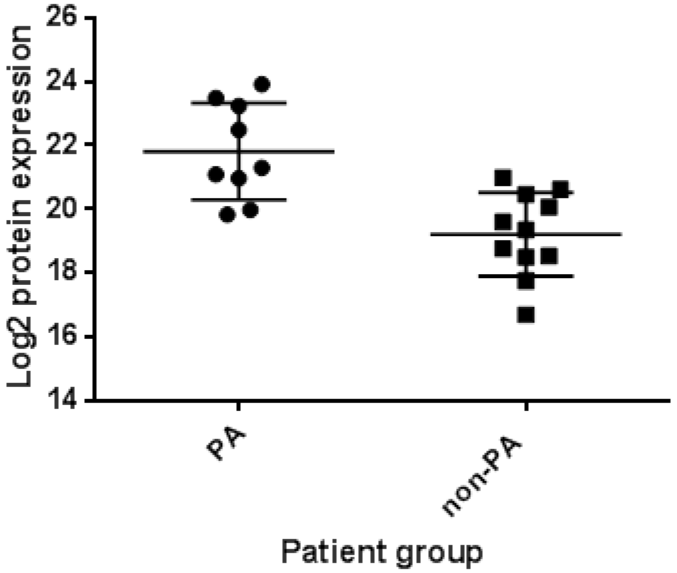

Figure E1 Comparison of Pseudomonas and Non-Pseudomonas groups in original proteomics screen (p=0.007)

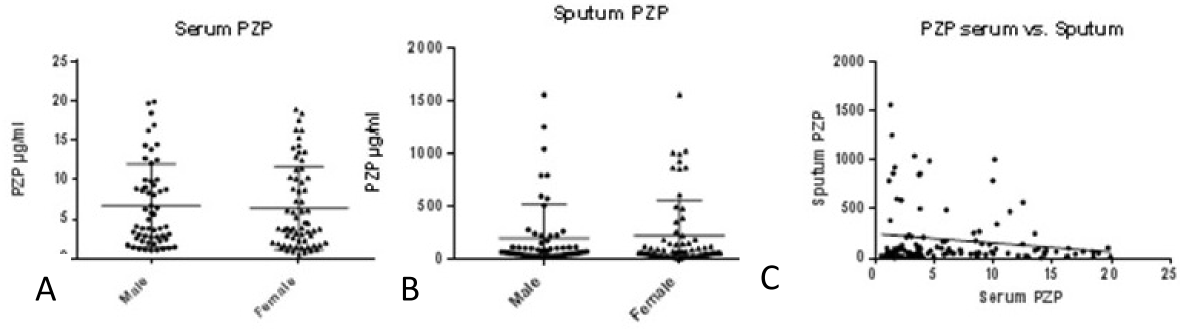

Figure E2 - A, Serum PZP in male and female patients (error bars show mean with SEM); B Sputum PZP in Male and Female patients (error bars show Mean and SEM); C, Correlation between serum and sputum PZP (p=0.07).

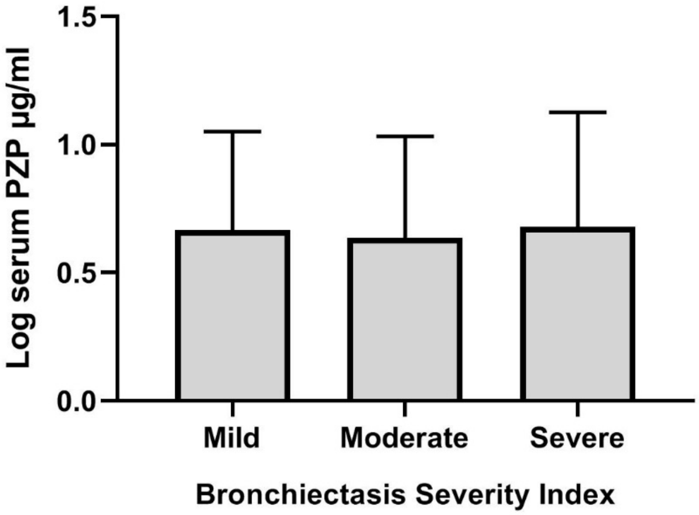

Figure E3 . Serum pregnancy zone protein and the bronchiectasis severity index. No relationship was observed between PZP and BSI (p=0.15 by ANOVA).

Variable	N (%) or mean (SD)
N	20
Mean Age	62 (12.5)
Sex	60% female
Mean FEV1 (% predicted)	60.77% (21.38)
Mean BSI	11.95 (4.53)

Organism cultured	
P. aeruginosa	8 (40%)
H. influenzae	4 (20%)
E. coli	1 (5%)
M. catarrhalis	1 (5%)
H. parainfluenzae	1 (5%)
S. maltophilia	1 (5%)
Achromobacter	1 (5%)
Enterobacter	1(5%)
Mixed flora	2(10%)

Table E2. Characteristics of the bacterial load study participants

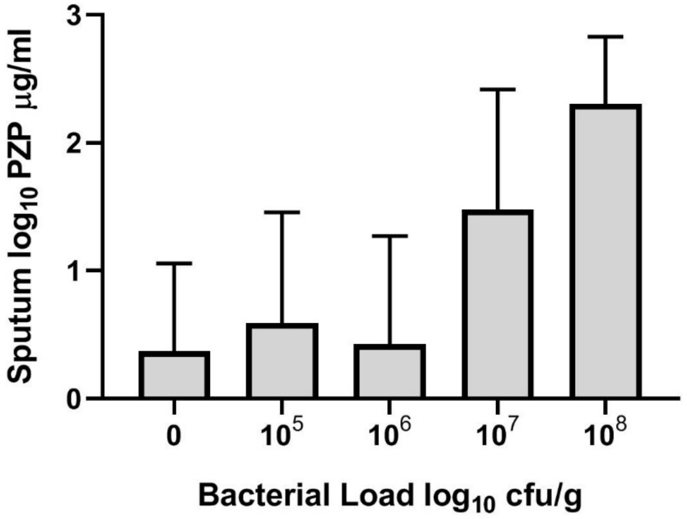

Figure E4. The relationship between bacterial load and sputum PZP excluding patients with *P. aeruginosa* infection. The difference across the groups is statistically significant by ANOVA $p<0.0001$.

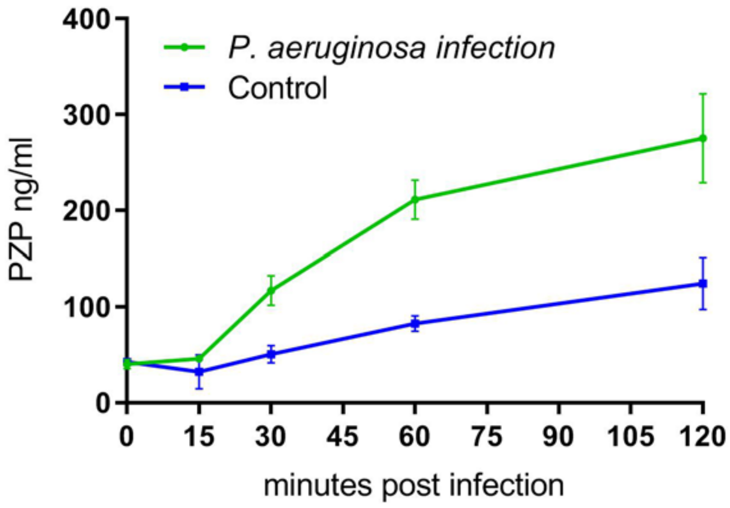

Figure E5. Release of PZP following *P. aeruginosa* infection. 10(6) neutrophils per time point were infected at a multiplicity of infection of 1:10. Following centrifugation to remove bacteria and neutrophils, PZP was measured in supernatant by ELISA. Results are the mean of 3 biological replicates.

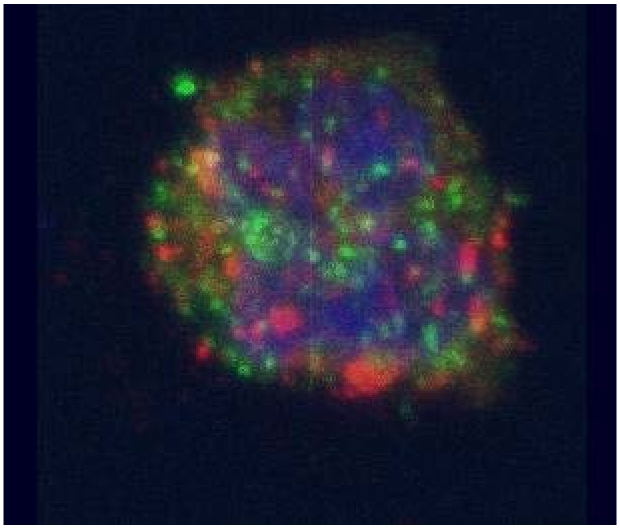

Figure E6 Microscopy image of neutrophils showing DNA: DAPI, Blue, MMP-9: Green and Pregnancy zone protein: Red.

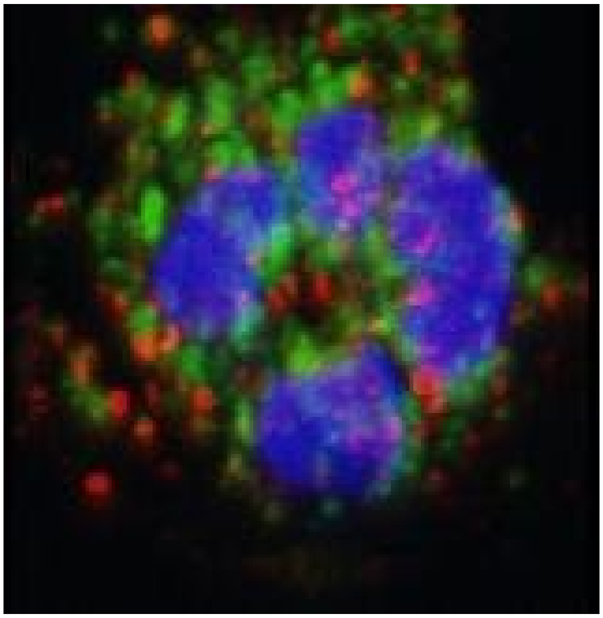

Figure E7 Microscopy image of neutrophils showing DNA: DAPI, Blue, MPO: Green and Pregnancy zone protein: Red.

3D PZP and MMP9.avi

Video E1 – 3D video of neutrophils stained for PZP and MMP9

3D PZP and MPO granules.avi

Video E2 – 3D video of neutrophils stained for PZP and MPO

N=40	
Mean Age (SD)	72.5 (8.72)
Sex	66.67% male
Mean FEV1 (% predicted)(SD)	68.78% (20)

Table E3. COPD study patient characteristics

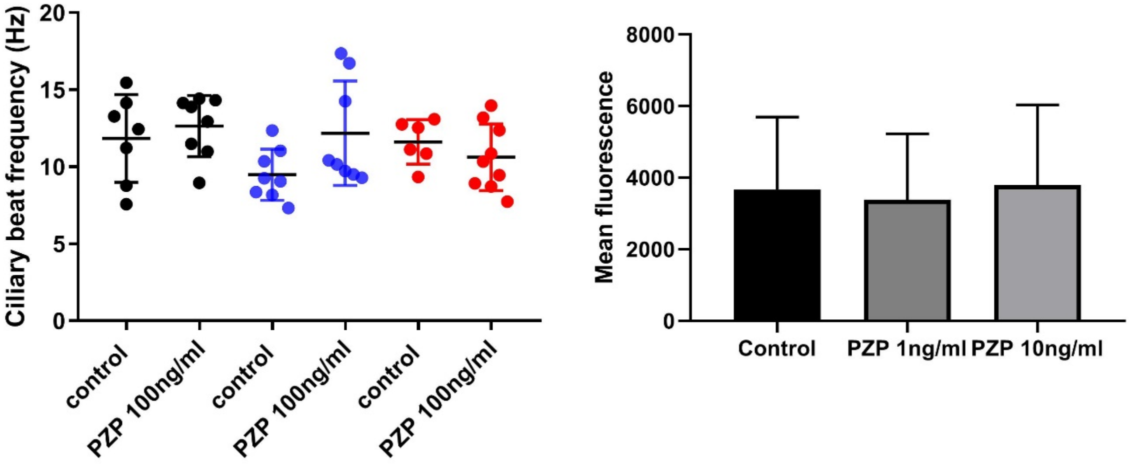

Figure E8. No effect of pregnancy zone protein at physiological concentrations on ciliary beat frequency (n=3 replicates from different donors, n=8 observations per donor). No significant differences were observed. Right shows no statistically significant difference in neutrophil phagocytosis after exposure to physiological doses of pregnancy zone protein (N=3 biological replicates from different donors, p=0.11).

Supplementary methods

Patients and clinical assessments

Patients were recruited at a specialist Bronchiectasis clinic at Ninewells Hospital, Dundee, UK. Inclusion criteria were: age ≥18 years, bronchiectasis confirmed by high resolution CT scan, chronic expectoration with ability to provide a sputum sample at the study visit and providing written informed consent. Exclusion criteria were: bronchiectasis due to cystic fibrosis, active allergic broncho pulmonary aspergillosis, active non-tuberculous mycobacterial infection, chronic use of oral corticosteroids and inability to provide informed consent. Ethical approval for the study was given by the East of Scotland Research Ethics Committee, approval number 12/ES/0059.

Patients underwent clinical evaluation including microbiology (described below), assessment of severity of disease using the bronchiectasis severity index, as previously described(13), and recording of exacerbation frequency. Exacerbations were defined as administration of antibiotics for increasing respiratory symptoms as defined by the British Thoracic Society(14). Sputum was obtained from all subjects during a period of clinical stability and split into whole (unprocessed) sputum for microbiology and sputum which was diluted 1:8 with PBS and then

centrifuged at 3000g for 15 minutes at 4°C . All sputum processing took place within 2 hours of expectoration and freeze thaw cycles were avoided. The supernatant was stored at -80°C until analysis.

Sputum protein profiling

Protein concentrations of sputum supernatants were quantified using Pierce 660 protein assay. Fifty micrograms of sputum protein from each sample was added to an equal volume of acetonitrile before incubating at 100°C for 15 min. The samples were dried down in a centrifugal vacuum and resuspended with 50mM ammonium bicarbonate (pH8.5) to a final concentration of 1 mg/ml. Samples were then reduced and alkylated before subjecting to nano-flow-LC-MS/MS analysis according to the previous report(15).

Protein identification and label-free quantification were carried out using Maxquant (version 1.4.1.2) against Uniprot-human database (version 2014-07-09). The fixed modification was carbamidomethylation on cysteine, and variable modifications include oxidation on methionine and N-terminal acetylation. FDR for protein identification was set to 1% at protein level. Data visualisation was carried out using SIMCA P (version 13.0.3). For statistical analysis, the dataset was

log2 transformed before subjecting to t test using Perseus (version 1.5.4.1). The Benjamini-Hochberg false discovery rate method was used and corrected p values of $p<0.05$ is considered significant.

Pregnancy zone protein ELISA

PZP was measured using a commercial ELISA kit (Cloud-Clone Corp., SEG324Hu and SEG324Mu). Validation was performed according to published recommendations(16). The optimal dilutions were 1:100,000 for sputum and 1:500 for serum and 1:5 for murine Broncho-alveolar lavage (BAL) samples.

All sputum samples used in ELISA were processed within 2 hours of expectoration. We confirmed stability of PZP in sputum by incubated sputum at room temperature for 48 hours with samples taken at 4, 24 and 48 hours as shown below (Figure S9)

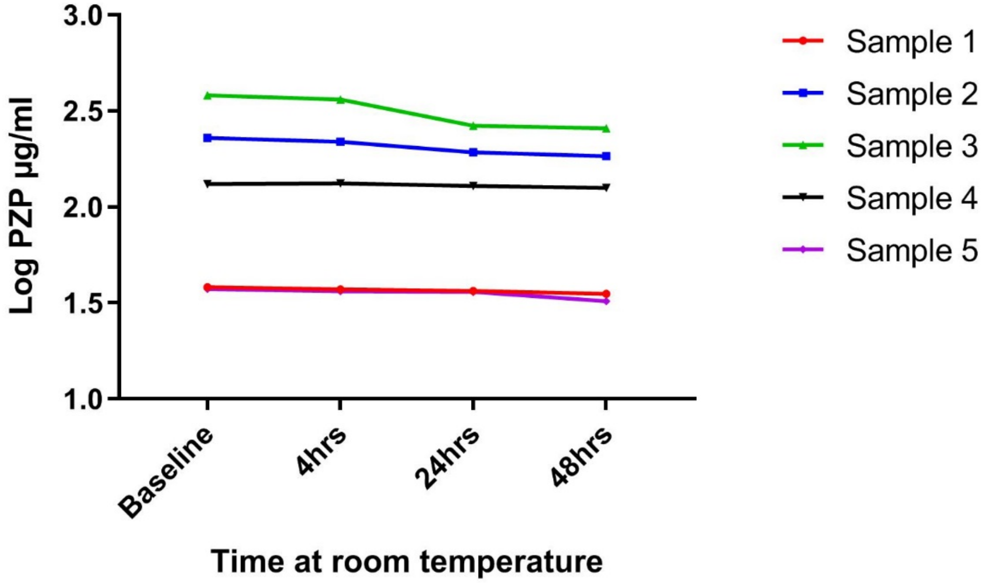

Figure E9. Stability of PZP in sputum over 48 hours. Samples were incubated in the laboratory at room temperature and aliquots processed for measurement of PZP (as described in the methods) at the indicated time points. No significant differences were observed comparing PZP at baseline and after 4 hours at room temperature (p=0.8)

Quantification of sputum NETs

Measurement of histone-elastase and DNA-elastase complexes provide a semi-quantitative assessment of neutrophil extracellular traps in sputum and assays were performed as previously described(17).

Leukocyte studies in healthy volunteers

Neutrophils and peripheral blood monocytes were isolated from healthy volunteers using Percoll Gradient Density Centrifugation as previously described(18). Neutrophils and monocytes were stimulated with Phorbol myristate acetate (PMA, 0.1-100µg/ml), N-formylmethionine-leucyl-phenylalanine (fMLP, 0.1-100µg/ml) and bacteria (*Eschericiacoli* strain BL21 and *P. aeruginosa* strain PA01, at multiplicity of infection ranging from 10:1 to 1:100. Following stimulation neutrophils were centrifuged at 1200g for 5 mins. PZP was measured in the resulting supernatant.

Immunofluorescence was used to confirm and localize PZP within neutrophils and NETs and identify co-localisation with other neutrophil proteins. Cells were seeded to glass coverslips ($2*10^5$ cells per slip) in 500µl RPMI (containing 2% human serum), for 1 hour. NET formation was induced by treatment for 4 hours with 600nM PMA.

Samples were fixed in 4% paraformaldehyde for 1 hour. Cells were permeabilized in 0.5% Triton X-100 for 1min before blocking with 5% BSA. Cells were labelled with primary antibodies, mouse monoclonal antibodies to PZP (Sigma-Aldrich HPA041471) and a rabbit polyclonal antibody to one of the following: neutrophil

elastase(R&D MAB91671), matrix metallopeptidase 9 (MMP9) (Thermo-Fisher MA5-14228), lactoferrin (Thermo-Fisher MA5-18107) or myeloperoxidase (MPO) (Thermo-Fisher MA1-80878) followed by appropriate secondary antibodies (goat anti mouse Alexafluor 488 and goat anti rabbit Alexafluor 594). Samples were stained with DAPI, mounted on clean glass slides and observed by confocal microscopy (Leica TCS SP5). Co-localisation of PZP with neutrophil granule proteins was quantified using Manders overlap coefficient which calculates the proportion of overlap of each channel with the other, with a value of 1 indicating perfect colocalization and 0 indicating no colocalization.

Electron Microscopy

Granulocytes isolated by Percoll gradient were fixed in 4% paraformaldehyde, suspended in an agar pellet and stored in 30% wt/vol sucrose until use. Cell pellets were frozen in cryomoulds using OCT(Tissue-Tek). 10µm cryosections were cut, blocked with BSA and labelled with anti-PZP antibody (Sigma-Aldrich HPA041471). To visualise the location of PZP by electron microscopy cells were labelled with a nanogold secondary (rabbit anti goat 1:200, EMS) followed by gold enhancement for 20mins (Universal Biologics 2114)

Labelled sections were fixed in 2.5% glutaraldehyde, post fixed in osmium tetroxide, dehydrated using a gradient of ethanols (70%, 90% and 100%) and embedded in araldite. 70nm sections were cut using an ultramicrotome (ultracut E, Leica) and stained post embedding using heavy metals (Uranyl acetate and lead citrate). Cells were visualised on a transmission electron microscope (Jeol 1400+) using a digital 4kX4k camera (AMT 16X, Deben Ltd, UK), appropriate negative controls were conducted.

Murine model of acute inflammation

In-vivo procedures were conducted according to the requirements of the United Kingdom Home Office Animals Scientific Procedures Act, 1986 and approved by the University of Dundee ethical review committee. Female 10-12 week old C57/B6 mice. *S. aureus* strain RN6390 was subcultured at 1:100 dilution from an overnight culture into fresh TSB medium. Cells were grown at 37°C with shaking until an OD_{600} of 0.5 was reached, before harvesting and washing three times in 1x PBS. Cells were finally resuspended in 1 x PBS. Mice were anaesthetized and infected intranasally with 25 µl of the bacterial suspension at an infecting dose of 3×10^8 cfu. At 24 hours post infection the trachea was carefully dissected, intubated and bronchoalveolar

lavage performed with 3 x 0.4ml PBS. BAL was centrifuged at 1300 rpm for minutes at 4°C. Supernatant was stored at -80°C and the cell pellet resuspended in 110µl media containing serum. 10µl cells were mixed with 10µl trypan blue and counted on a haemocytometer. Cytospins were prepared and stained for differential cell counts using Wright-Giemsa staining kit. Murine PZP was measured using ELISA.

Sputum bacteriology

Quantitative bacterial culture was performed on sputum samples within 4 hours of expectoration. Sputum was homogenized in 50:50 volume 0.1% dithiothreitol and serially diluted in sterile 0.9% sodium chloride for plating on blood agar and choclate with bacitracin agar. Plates were inoculated with 100ul of diluted sample and colony forming units of each identified pathogen counted after 48 hours incubation. Bacterial density is expression as log10 cfu/g.

Microbiota sequencing

The AllPrep DNA/RNA Mini kit was used to extract DNA and RNA from whole sputum: Sputum was incubated in an equal volume of 1:10 diluted Sputolysin

(Calbiochem) for 30mins at 37°C, mixed with Buffer RLT as per the AllPrep kit protocol, then passed through QIAshredder columns (QIAGEN) with the supernatant undergoing sequential DNA and RNA extraction. extraction on the QIAcube automation platform. Alternatively, 0.1g whole sputum was incubated with Proteinase K for one hour at 55°C then processed as described in Zymo Quick-DNA Miniprep Plus solid tissues protocol, eluting into 50µL elution buffer. We assessed quality and quantity of the DNA and RNA by Nanodrop and Qubit machine, using the Qubit dsDNA broad range kit (Thermo Scientific). Metagenomic sequencing of the bacterial 16S rRNA gene was performed following the protocol in the Illumina library prep guide (https://www.illumina.com/content/dam/illumina-support/documents/documentation/chemistry_documentation/16s/16s-metagenomic-library-prep-guide-15044223-b.pdf), using primers targeting the V3 and V4 region.

Nextera XT Indices were added to each sample to allow multiplexing and the libraries sequenced using 2 x 300 paired end sequencing on the MiSeq platform using a MiSeq V3 kit (Illumina). Following sequencing, FastQ files were imported into QIIME (version 1.9.0) and quality of reads checked. Any reads with a Phred quality score less than Q20 were excluded when paired end reads were joined

together for each sample. Un-joined reads were excluded from subsequent analysis. Sequences were clustered into operational taxonomic units (OTUs) based on 97% sequence similarity using the UCLUST algorithm , aligned against the Greengenes Core reference alignment (Version 13.8) using PyNAST (Version 1.2.2). Taxonomy of the OTUs was assigned using the Ribosomal Database Project Classifier (Version 2.2) with the *de novo* OTU picking option (E10). OTUs were examined to remove singletons and unassigned OTUs, or OTUs identified as Eukaryota, Human and Cyanobacteria. The data was normalised to the lowest number of OTUs and the Shannon-Wiener Species Diversity Index (SWDI) of the samples determined.

Analysis was performed in QIIME. To compare groups patients were split into those with predominant (>50% OTUs) proteobacteria and those with predominant firmicutes at the phylum level as previously described.

<u>Antibiotic response study</u>

Patients were asked to attend the research centre if they developed symptoms of an exacerbation. Patients were reviewed by a physician and those who were prescribed antibiotics for a protocol defined exacerbation were included in the

study. Patients received treatment for 14 days based on their previous sputum microbiology. Spontaneous sputum samples obtained as baseline and after 14 days were used for PZP measurement.

COPD cohort study

To compare sputum PZP levels in bronchiectasis to those from patients with COPD, 40 patients with COPD without underlying bronchiectasis were enrolled while clinically stable (4 weeks free from antibiotic or corticosteroid therapy). Spontaneous sputum samples were obtained and processed in the same way as the bronchiectasis samples with sputum PZP and sputum NETs measured by ELISA.

Neutrophil phagocytosis assay

Was evaluated by flow cytometry on a BD Fortessa. FITC-labelled *P. aeruginosa* strain PA01 were opsonised with 10% serum and incubated with neutrophils (multiplicity of infection 10:1) which had been treated with recombinant PZP at the indicated concentrations or vehicle control. Phagocytosis was quantified by the mean fluorescence after gating on the neutrophil population.

Ciliary beat frequency measurement

Primary nasal epithelial cells from healthy donors were obtained by brushing of the inferior nasal turbinate followed by culture at air liquid interface. Ciliary beating analysis and quantification was performed by high-speed videomicroscopy.
